# Multi-cultural cities reduce disadvantages in recognizing naturalistic images of other-race faces: evidence from a novel face learning task

**DOI:** 10.1038/s41598-022-11550-9

**Published:** 2022-05-27

**Authors:** Xiaomei Zhou, Catherine J. Mondloch, Sarina Hui-Lin Chien, Margaret C. Moulson

**Affiliations:** 1 Department of Psychology, Toronto Metropolitan University, 350 Victoria St, Toronto, M5B 2K3 Canada; 2 Department of Psychology, Brock University, St Catharines, L2S 3A1 Canada; 3 Graduate Institute of Biomedical Science, China Medical University, , https://gibs.cmu.edu.tw/

**Keywords:** Psychology, Human behaviour

## Abstract

People often find it more difficult to recognize other- than own-race faces. This other-race effect is robust across numerous ethnic groups. Yet, it remains unclear how this effect changes in people who live in a multiracial environment, and in immigrants whose lifetime perceptual experience changes over time. In the present study, we developed a novel face recognition test that approximates face recognition in the real world. We tested five groups of White and East Asian adults (n = 120) living in racially homogeneous versus heterogeneous cities and East Asians who immigrated to a multiracial city between infancy and adulthood. Multiracial cities reduce the other-race effect. The magnitude of the other-race effect changes as a function of experience, mirroring the racial diversity in perceivers’ living environment. Our study highlights the challenge of forming reliable face representations across naturalistic facial variability and suggests a facilitative role of multiracial environments in eliminating the other-race effect.

## Introduction

In many countries around the modern world, successful social interactions require people to recognize individuals from different ethnic groups. Yet not all faces are equally recognizable. The other-race effect (ORE) describes the phenomenon whereby adults show more difficulties in recognizing and memorizing faces of a different race than faces of their own race^1^. Emerging during infancy, the other-race effect is robust across a broad range of research paradigms and participant populations^2^. Consequences of the other-race effect are severe. It can cause social embarrassment^3^, formation of race-specific stereotypes, and is associated with the emergence of implicit racial bias^4^. It also makes cross-race eyewitness identification highly unreliable, leading to serious miscarriages of justice^5^.

Research over the past decade has characterized the ORE. Although it remains debatable whether the development of the ORE follows a perceptual narrowing or perceptual learning process^6–8^, researchers generally agree that asymmetrical perceptual experience with own- and other-race faces contributes to the phenomenon (see also social-cognitive explanations of the ORE^9^). According to this experience-based view, a lack of perceptual experience with other-race faces, as a result of person-environment interaction, leads to differences in processing, representing, memorizing, and learning own- versus other-race faces in adulthood. Adults show reduced sensitivity to differences among other-race faces in featural shape and spacing^10–12^, impairments in norm-based coding^13^, and in encoding, consolidation, and/or retrieval of other-race faces in visual working memory as compared to own-race faces^14^. Whether adults process other-race faces less holistically is debated^11,15–18^.

Despite decades of research on the ORE, past studies suffer two important limitations: (1) face images have not incorporated naturalistic variability in facial appearance; and (2) variability in experience with other-race faces has largely been ignored. We addressed both limitations in the current study. In most studies, researchers have represented an individual’s appearance with a single image or a limited number of highly controlled images that varied on only one or two dimensions (e.g., head orientation, illumination). For example, in the classic Cambridge Face Memory test (both CFMT-Original and CFMT-Chinese)—one of the most widely used tests assessing White and East Asian perceivers’ face memory^19,20^—face images incorporate limited naturalistic facial variability. Only two dimensions, lighting conditions and head orientations, vary across face images; other dimensions are either eliminated (e.g., facial outline, facial hair, accessories, make-up) or tightly controlled (e.g., background, expressions). Neglecting within-person variability in appearance underestimates the challenge of face identification in daily life^21–24^; faces viewed in the real world vary naturally in appearance due to changes in lighting, expressions, viewing angles, hairstyle, and make-up. Thus, the pictorial cues (e.g., luminance, shadows) that participants rely on in laboratory tests become less reliable in daily life. This oversight leaves a significant gap in knowledge about the magnitude of the ORE in the real world.

Second, the ORE has been primarily tested in populations who live in more racially homogeneous environments (i.e., those who typically receive low other-race contact and therefore sit on the end of the perceptual experience distribution^10,11,20^). Consequently, the core prediction of the perceptual experience hypothesis—that the magnitude of the ORE changes with perceptual experience—lacks convincing empirical evidence. Whereas a number of studies reported a positive correlation between self-reported interracial contact and performance for other-race faces in discrimination and recognition tasks^25–29^, others found no such correlation^30–33^. Two meta-analyses reported that self-rated interracial contact accounts for only 2% of the variability in the magnitude of the ORE^1,25^. Few studies compare the ORE in groups with different perceptual experience with own- and other-race faces. For example, there are studies comparing the ORE in individuals who live in racially integrated or segregated environments^34,35^. Other studies investigate how the ORE changes when individuals’ race-related experience changes as a result of immigration^12,36–39^, or interracial adoption^40^. These studies provide evidence that increased other-race contact helps to reduce the ORE. Nonetheless, variations in research paradigms, face stimuli, and participant populations make it difficult to generalize results. A more systematic examination of the nuances of perceptual experiences is needed to better elucidate how different levels of perceptual experience shape the magnitude of the ORE.

To address these gaps, we designed two versions of a novel face memory test: the White Variability Face Memory Test (VFMT-White) and East Asian Variability Face Memory Test (VFMT-EA), using the structure of the classic Cambridge Face Memory Test (CFMT). Both versions of the test include stimuli that incorporate a wide range of naturalistic within-person variability. We then tested the magnitude of the ORE in five groups of white and East Asian participants, living in three cities, with different lifetime histories of perceptual experience with own- versus other-race faces. Participants included White and East Asians who were born and raised in racially homogeneous environment, East Asians who were born and raised in one of the most multiracial areas in the world, the Greater Toronto Area (GTA), and East Asians who immigrated to the GTA at different ages between infancy and adulthood. We systematically investigated: (a) how the ability to form robust face representations across facial variability differs for own- and other-race faces, and (b) how different lifetime histories of perceptual experience shape the magnitude of the ORE in adulthood. Answering these questions will help inform a cross-culturally valid theoretical account of the relation between perceptual experience and the recognition of own- versus other-race faces.

## Method

### Participants

Participants were 120 White and East Asian adults between 18 and 39 years from Toronto Metropolitan University in Toronto, Brock University in St. Catharines and China Medical University in Taichung. Specifically, this sample comprised five groups of 24 participants based on their ethnicity, city of residence, and lifetime history of perceptual experience: (1) White adults living in St. Catharines, a predominantly white city in Canada (19 female, *M*_age_ = 22.67, *SD* = 4.25, age range = 18–39); (2) White adults born, raised, and currently living in the Greater Toronto Area (GTA; 22 female, *M*_age_ = 19.67, *SD* = 4.00, age range = 17–37); (3) East Asians who were born, raised, and currently living in the GTA (17 female, *M*_age_ = 19, *SD* = 1.41, age range = 17–23); (4) East Asian immigrants to GTA, Canada (13 female, *M*_age_ = 24.21, *SD* = 4.68, age range = 17–32); and (5) East Asian adults (13 female, *M*_age_ = 22.54, *SD* = 3.09, age range = 19–28) born, raised, and currently living in Taichung, an East Asian predominant city. All East Asian immigrants were born in East and Southeast Asian countries (i.e., China, South Korea, Philippines) and immigrated to the GTA for educational and career purposes. The average age of East Asian immigrants moving to Canada was 20.21 years old (median = 23.25, mode = 26, *SD* = 7.45, Range = 1–28), with an average length of their stay in Canada of 49.30 months (4.11 years, median = 35.5 months, mode = 9 months, Range = 9–204 months, *SD* = 53.89). Of the East Asian immigrants, 4.17% arrived during infancy (0–2 years, *n* = 1), 8.33% in childhood (3–11 years, *n* = 2), 16.67% in adolescence (12–17 years, *n* = 4) and 70.83% in adulthood (> 18 years, *n* = 17). Fifteen additional participants were tested but excluded from final data analyses because of program or experimenter error (*n* = 5), reported familiarity with the learning models (*n* = 5) or incomplete demographic questionnaire (n = 5). To ensure that our White in St. Catharines group and EA in Taichung group had minimum interaction with other-race individuals, we also excluded an additional 19 participants from these two groups who reported having lived in countries with large populations of other-race individuals for more than 3 months (n = 6), having other-race relatives (n = 4), or having lived with an other-race individual (n = 9). All participants gave written informed consent and received either research credit or a small honorarium for their participation.

According to Statistics Canada census data^41^ in 2016, St. Catharines is the sixth largest urban area in the province of Ontario, Canada, with a total population of 133,113 in 2016. In St. Catharines, 84.6% of the population is White and 2.7% is East Asian (including 2.1% Chinese, 0.4% Korean and 0.2% Japanese). The Greater Toronto Area (GTA) is the most populous and multicultural metropolitan area in Canada, and the fourth most populous municipality in North America. In 2016, the total population of the Toronto Census Metropolitan Area was 5.93 million; 47.8% of the population is White and 12.4% are East Asian (including 10.8% Chinese, 1.2% Korean, and 0.4% Japanese). According to the Ministry of the Interior National Immigration Agency in Taiwan^42^, Taichung is the second most populous Taiwanese city with a population of 2.82 million people in 2019. Less than 0.2% of the total population in Taichung are White residents. According to the census data, these characteristics reflect the demographic nature of the area from which the sample was drawn.

An a priori analysis indicated that our experimental design required 80 participants to uncover an effect size of *f* = 0.25 in a 2 × 5 mixed ANOVA based on an alpha of 0.05 and 95% power (correlation among repeated measures = 0.5, no nonsphericity correction). We purposely over-sampled with a goal of testing 24 participants per living group, anticipating that some participants would not be included in the final analysis, and data collection continued until this goal was reached. Our final sample size (n = 120), after excluding data from participants as described above, exceeded the sample size required as indicated by the a priori power analysis.

### Stimuli

Consistent with the classic Cambridge Face Memory Test (CFMT), six white and six East Asian female celebrities were chosen as targets, and forty-six white and forty-six East Asian female celebrities were used as distractors. For each race, eight photographs of each target (two photos showing left profile, three showing frontal profile, and three showing right profile) and three photographs of each distractor (each showing a different head orientation) were taken from the Internet via a Google image search.

**Figure 1:**
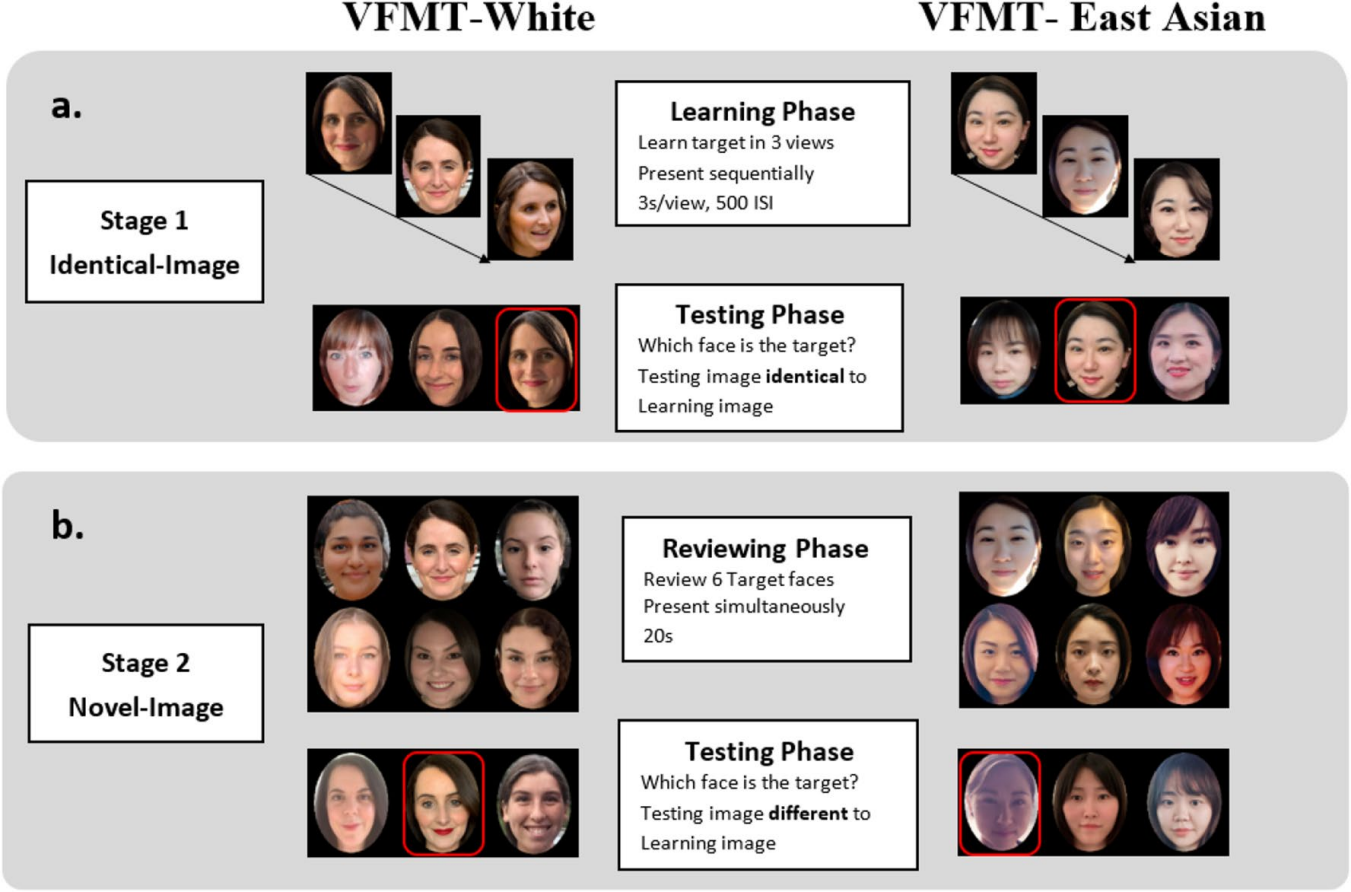
Demonstration of targets’ and distractors’ face stimuli and the structure of the two face memory tasks: VFMT-White (left) and VFMT-East Asian (right) for Stage 1 (**a**) and Stage 2 (**b**). Target’s faces in testing phases were circled in red. Note that only left view of models for Stage 1 and frontal view of models for Stage 2 were shown in the figure for demonstration purposes, but all 3 views (left, frontal and right) of models were presented in the actual testing for each testing phase. (Copyright restrictions prevent publication of the original images used in the experiment. Images are illustrative of the experimental stimuli and depict individuals who did not appear in the experiments. We obtained written informed consent for publication of identifying images in an online open-access publication).

Different from the CFMT-original, we selected ambient images of faces that incorporate extensive naturalistic facial variations, including not only variation in lighting conditions and head orientations (like CFMT), but also variation in expressions, hairstyles, accessories, and make-up (see Fig. 1). We did not control for the specific angle of the models’ face looking toward the left or right direction (i.e., 1/3 left or right profile in CFMT); instead, we included photographs of models having a more general left or right head orientation as long as both models’ left and right eyes were visible to perceivers. Celebrities’ faces were used because it was more feasible to access an ample number of face images of 104 White and East Asian identities. The celebrities (see Supplementary Material for details) were chosen because they were well known in the country of origin, but unfamiliar to the Canadian and Chinese participants tested in the experiment. Across each race, models were matched for age, and faces presented in each testing pair were physically similar to each other (see Table 1 for target and distractor models’ age). For each person, we selected the first eight (for targets) or three (for distractors) photos in which their face was equal to or larger than 150 pixels in height and was visible with no obstructions (e.g., sunglasses). This resulted in a total of 372 images downloaded. The images were then cropped so that only the face and part of the neck were displayed and were resized to 75 × 90 mm. At the approximate viewing distance of 60 cm, images subtended 7.15° (width) × 8.58° (height) of visual angle.

**Table 1:** Statistics of targets’ and distractors’ age.

Race	Identities	M	SE	Median	Mode	Range	95% CI
White	Targets (n = 6)	35.67	1.43	36	37	31–41	[32, 29]
	Distractors (n = 46)	30.54	0.67	30	30	18–40	[29, 32]
East Asian	Targets (n = 6)	32	1.06	31.5	30	30–37	[29, 35]
	Distractors (n = 46)	31.13	0.80	32	33	5.39	[30, 33]

### Procedure

All experimental protocols were approved by the Human Research Ethics Committee of Toronto Metropolitan University, and the studies were carried out in accordance with guidelines of the ethics review board at Toronto Metropolitan University, Brock University and China Medical University. Informed consent was obtained from all participants.

The Variability Face Memory Test (VFMT) follows the same format and procedure as the original CFMT (Duchaine & Nakayama, 2006). Participants’ face memory was tested for both White and East Asian faces, with the order of face race counterbalanced across participants, such that half of the participants were tested with own-race faces first, and the other half with other-race faces first. Prior to the actual task, participants completed three practice trials with cartoon faces to gain familiarity with the testing procedure. Within each race, the Variability Face Memory Test (VFMT) comprised two stages: the Identical-Image Stage and the Novel-Image Stage.

Upon completion, participants filled out a familiarity and racial contact questionnaire. In the questionnaire (see Supplementary Material), participants were asked to indicate whether they were familiar with any of the faces, and to provide relevant semantic information about any person recognized (e.g., name, occupation or any other identifying information). They also answered questions assessing their contact with other-race identities (i.e., White adults contact with East Asian identities and vice versa). For example, they were asked how many of their closest 10 friends were of East Asian/White ethnicity and how much current (e.g., I interact with Asian people on a daily basis) and previous experience (e.g., I went to high school where I interacted with Asian students) they had with other-race individuals. Specifically, they indicated the extent to which they disagreed/agreed with 12 statements regarding their interactions with White and East Asian people using a Likert scale of 1 (very strongly disagree) to 6 (very strongly agree).

#### Practice

In the practice trials, participants were instructed to sequentially memorize 3 images of Bart Simpson with each image presented for 3 s and showing a different viewpoint (left 1/3 profile, a frontal view, and a right 1/3 profile). In the learning phase, participants always learned left, frontal and then right profile. Participants were then presented with one of the learned images of Bart Simpson (target) along with two other cartoon faces (distractors) and were asked to pick out the target (Bart Simpson) from the two distractors by pressing one of the number keys (“1,” “2,” or “3”) shown right below each of the three faces in the testing. They were then asked to pick out the second and third learning images of Bart Simpson, each of which was presented along with two new distractors. Participants moved to the actual face memory test after they correctly identified all three images of Bart’s face in the practice (i.e., 100% accuracy). If they failed, the practice stage started over. 97.5% of participants (*n* = 117) successfully completed the practice phase on their first attempt, and 2.5% (*n* = 3) failed on their first attempt but passed on their second attempt.

#### Identical-image stage

The structure of the Identical-Image Stage was similar to the practice stage. Participants were instructed to learn six target identities. Each target was learned with three images that varied in pose and incorporated a wide range of naturalistic within-person variability (e.g., different expressions, lighting, hairstyle, make-up). Each image was presented for 3000-ms with a 500-ms inter-stimulus interval (ISI). Following presentation of each view, each learned image was presented among two distractors and participants were asked to indicate which face was the target (see Fig. 1). This step was then repeated for each identity until all six targets were learned. This resulted in a total of 18 trials (3 views × 6 target identities). Test faces were presented until participants pressed a key to respond. Because the testing image was identical to learning image, participants could use pictorial cues available in the test (information specific to a particular image, such as specific shadows) to guide their recognition.

#### Novel-image stage

Following the Identical-Image Stage, participants were presented with a single review image showing a frontal view of each of the six target faces (i.e., the previously learned middle view of the targets; see Fig. 1b. Reviewing Phase) and were given 20 s to review all learned six target identities. Following the review stage, participants were presented with 30 trials (5 presentations × 6 targets) in a fixed, random order such that the order of the viewpoints (left, frontal, right) for each target and the presentation order of the six targets were randomized across the 30 testing trials. On each trial, a novel image of a target was presented along with two novel distractor faces. Participants’ task was to identify the learned target from the distractors. Notably, the novel images incorporated a wide range of natural facial variability. Participants were instructed that they were to recognize the same target identities learned during the Identical-Image Stage but that new images of each identity would be presented.

Throughout the Identical- and Novel-Image Stages, it was made clear that some distractor images were presented in more than one trial, meaning that both old and novel images of the same distractors were used, therefore, participants could not rely on an impression of looking ‘new’ to make familiar or unfamiliar judgments. The number of distractor images repeated, and how often they were repeated, was matched to the trial-by-trial structure of the original CFMT. The original CFMT has a third *Novel Images with Noise* Stage. Given the difficulty of the VFMT due to the wide range of variability present in the images, adding noise to novel images of even own-race faces would likely result in a floor effect. Therefore, the *Novel Images with Noise* Stage was not included in the current version of the task.

The VFMT task was programmed and tested using MATLAB. The Matlab codes for the VFMT are available from the Corresponding Author, X. Zhou.

## Results

### Personal contact with own- and other-race individuals

**Figure 2:**
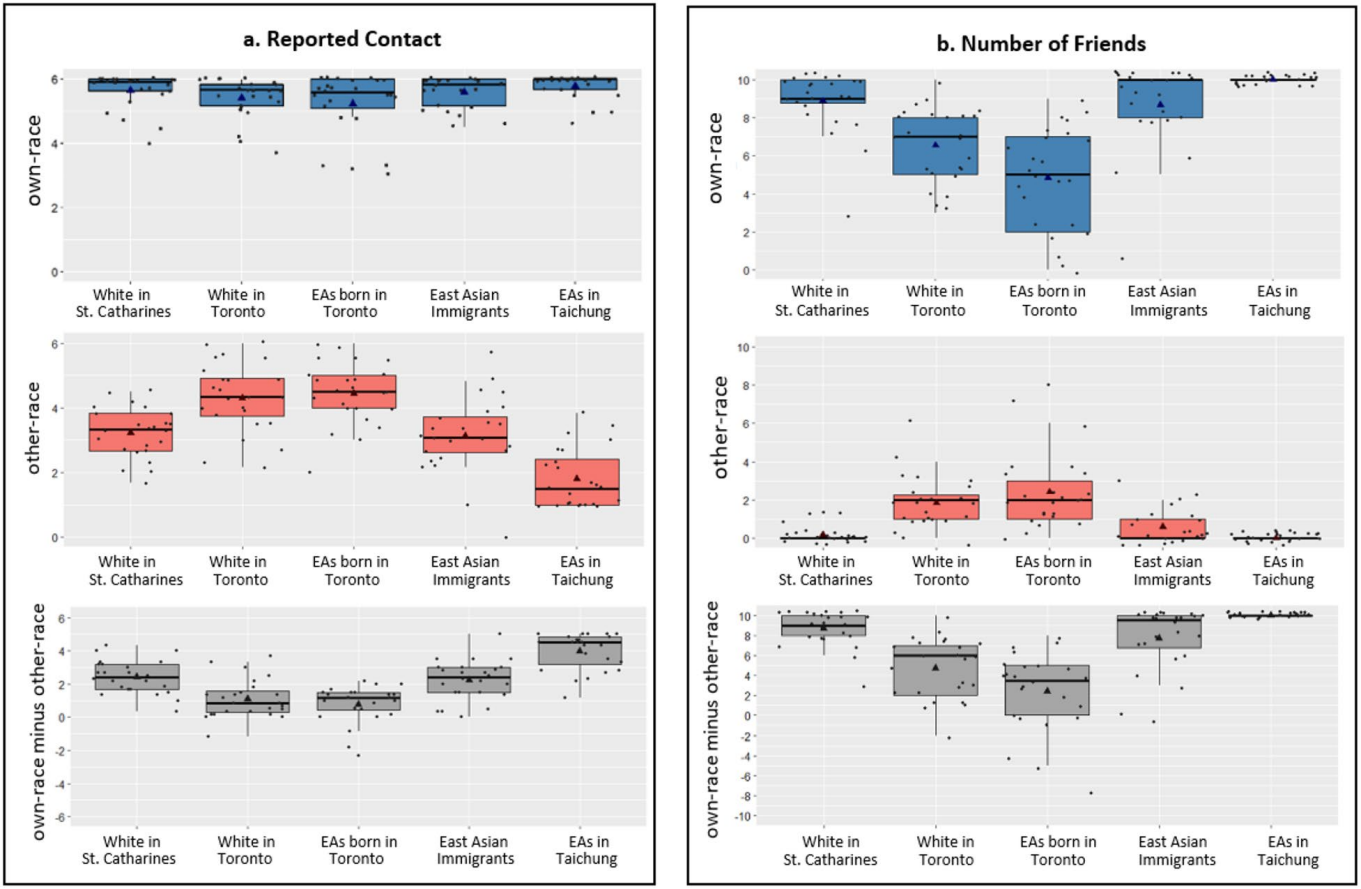
Box-whisker plots showing (**a**) reported own- versus other-race contact and the difference between own- and other-race contact (left panels) and (**b**) reported number of own- versus other-race friends, and the difference between own- and other-race friends (right panels) in the five groups of participants. Midline and triangle inside each box represent medians and means, respectively. The bottom and top of boxes indicate the 1st and 3rd quartiles. Black dots represent contact (left panels) or number of friends (right panels) reported by individual participants.

In addition to the group manipulation, data from the contact questionnaire confirmed the expected variability in personal contact with own- and other-race individuals in the five groups of participants. A 2 (own vs. other-race contact) × 5 (participant groups) mixed ANOVA revealed a significant interaction between race of contact and participant group, *F*(4,114) = 31.04, *p* < .001, *η*_p_^2^ = 0.52. We then decomposed the interaction by looking at how own-race contact versus other-race contact differs separately in the five participant groups (see Fig. 2). Significant effects were followed up with Bonferroni-corrected pairwise comparisons. All groups reported a high level of interaction with own-race individuals (*M* = 5.53, *SE* = 0.06; out of 6). EAs born in Toronto group reported having less own-race contact than EAs in Taichung group, *p* < .041. All other groups did not differ in their reported own-race contact, *p*s > 0.280. In contrast, other-race contact was lowest in Taichung EAs group (*M* = 1.81, *SE* = 0.19, *p*s < .001), highest in Toronto born EAs group (*M* = 4.44, *SE* = 0.20, *p*s < .001) and Toronto born White group (*M* = 4.31, *SE* = 0.22, *p*s < .004), and intermediate in White in St. Catharines group (*M* = 3.23, *SE* = 0.16, *p*s < .004) and EA immigrant group (*M* = 3.13, *SE* = 0.24, *p*s < .002). Reported other-race contact did not differ between the Toronto born EA group, and Toronto born White group, *p* = 1.000. And other-race contact also did not differ between the White in St. Catharines group, and Toronto EA immigrant group, *p* = 1.000. We then calculated the difference between own- and other-race contact and ran a one-way ANOVA to compare how the contact difference differs across the five groups. The Toronto born White and East Asian groups had lower own-versus other-race contact differences than both the White in St. Catharines and EA immigrant groups (*p*s < .004), and the EA in Taichung group (*p*s < .001).

In addition, a mixed ANOVA analysis on the number of friends also revealed a significant interaction between race of friends and participant group, *F*(4,114) = 32.30, *p* < .001, *η*_p_^2^ = 0.53. All groups reported having more than 4.8 own-race friends (*M* = 7.77, *SE* = 0.24), with EAs born in Toronto group reported having the smallest number of own-race friends (*M* = 4.83, *SE* = 0.55, *p*s < .027). In contrast, the White in St Catharines, EA immigrants, and EAs in Taichung groups reported having similar number of other-race friends, *p*s > .923, and these groups reported having a smaller number of other-race friends than both Toronto born White and EA groups, *p*s < .007 (see Fig. 2). The difference between the number of own- and other-race friends were the smallest in Toronto born White and EA groups (*p*s < .039), highest in the EAs in Taichung group (*p*s < .001) and intermediate in the White in St. Catharines and EA immigrant groups (*p*s < .001). In summary, as expected, whereas individuals who were born and raised in a multiracial environment (i.e., Toronto) tend to report having more comparable own- and other-race contact, individuals who live in a racially homogeneous environment tend to report having more contact with own-race individuals than other-race individuals.

### Sensitivity of VFMT-White and VFMT-East Asian

Next, we examined whether each race version of the Variability Face Memory Tests (VFMT) provides a sensitive measure of individuals’ face memory. For each race version, there were 18 testing trials in the first stage, and 30 testing trials in the second stage, resulting in a total of 48 trials. Therefore, stage 1, stage 2, and the two stages combined were scored out of 18, out of 30, and out of 48, respectively. Below, we report the normality and frequency distribution of each test. We tested five groups of participants who have very different levels of perceptual experience with White and East Asian individuals during development. Some found the VFMT-White more difficult while others found the VFMT- East Asian more difficult, providing us with a unique opportunity to examine the sensitivity of the VFMT with a large spread of scores and a full cross-over comparison.

**Figure 3:**
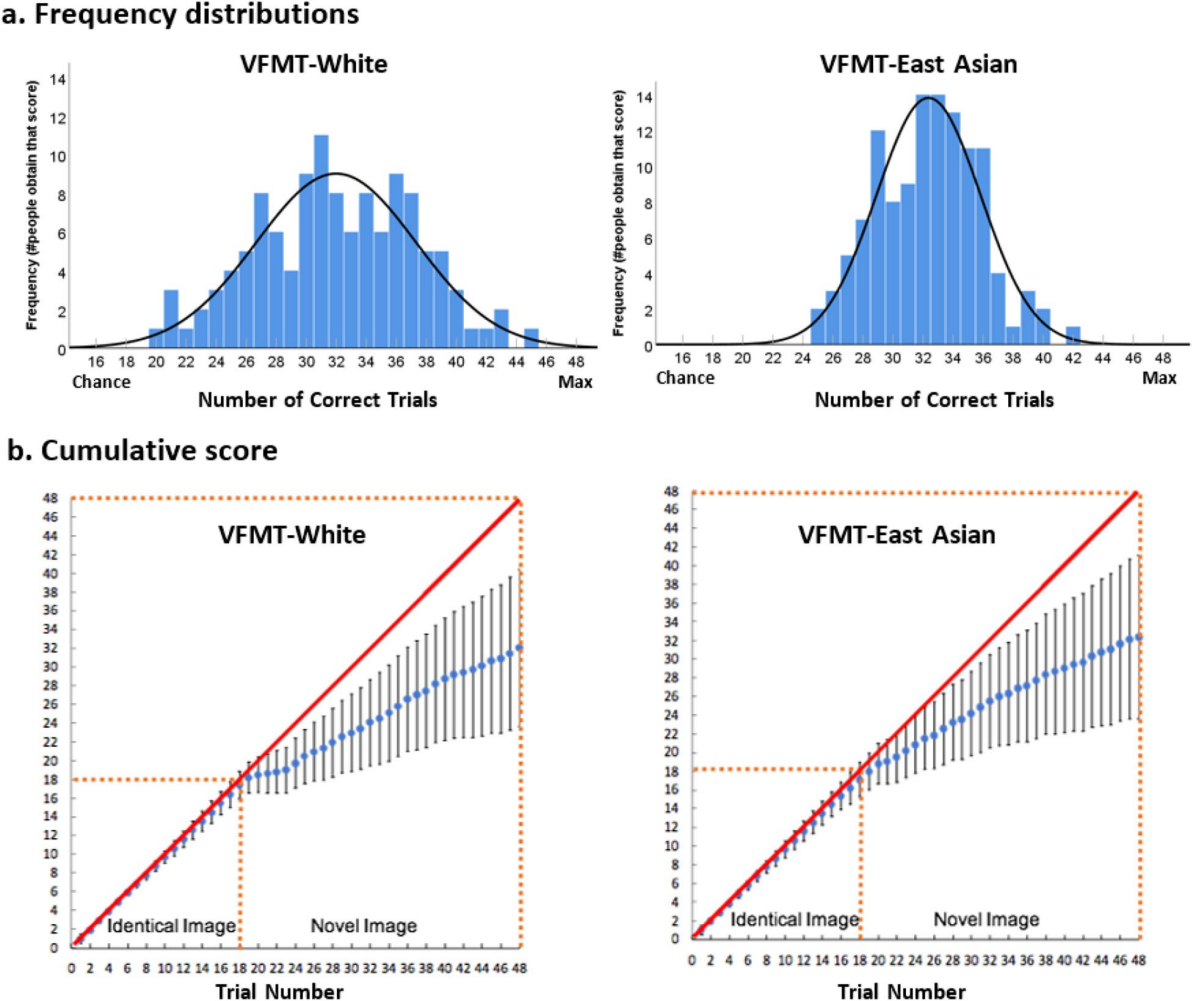
(**a**) Frequency distributions for the VFMT-White and VFMT-East Asian with best Gaussian fits to the data of 120 individuals. X axis indicates the number of correct trials, with chance level = 16 (i.e., 33.3% correct), and the maximum number of correct trials = 48(i.e., 100% correct). Y axis indicates the number of people that got the corresponding number of trials correct) (**b**) Average cumulative scores across 48 test items for White faces and Asian faces. The red diagonal lines indicate perfect performance (100% accuracy), and the blue dots indicate the average cumulative score at each test item, representing participants’ actual performance. Error bars indicate the standard deviation for the cumulative scores. The task is divided into two stages, with the first 18 items in the Identical-Image Stage, and last 30 items in the Novel-Image Stage.

Normality tests suggested that scores for VFMT-White and VFMT-East Asian were normally distributed (Kolmogorov–Smirnov statistic = .007, *df* = 120, *p* = .200, and Kolmogorov–Smirnov statistic = .076, *df* = 120, *p* = .084 for White and East Asian faces respectively). Data from both tests showed no evidence of skewness (White: Skew = − 0.053, *SE* = 0.22, *z* = − 0.24; Asian: Skew = 0.104, *SE* = 0.22, *z* = 0.47) or tailedness (White: Kurtosis = -0.46, *SE* = 0.44, *z* = 1.05; Asian: Kurtosis = -0.26, *SE* = 0.44, *z* = 0.58). There is a broad range of scores for both race versions of the test (VFMT-White: range = 20–45; VFMT-Asian: range = 25–42), making each test suitable for testing face memory in a typical adult population (see Fig. 3a). Figure 3b shows the average cumulative scores across the 48 test items along with the standard deviation. For both VFMT-White and VFMT-East Asian test, the average slopes (*m*) of the cumulative score for the Identical-Image Stage (White: *m* = 0.97; East Asian: *m* = 0.95) are larger and steeper than that of the Novel-Image Stage (White: *m* = 0.49; East Asian: *m* = 0.51), hinting that the Identical-Image Stage might be easier than the Novel-Image Stage.

### Recognition Accuracy for own- versus other-race faces

One-sample t-tests revealed that all accuracies were greater than chance level (33.3%), *p*s < .001, suggesting that poor scores are meaningful indicators of participants’ performance rather than floor effects. Descriptive statistics for recognition accuracy are shown in Table 2.

**Table 2:** Recognition accuracy statistics.

Stages	Race of faces	M	SE	Median	Mode	Range
Two stages combined	Own-race	70.32	0.82	70.63	75.00	45.50–94.00
	Other-race	63.65	0.82	64.38	67.63	41.75–89.38
	Total	66.98	0.69	67.50	67.63	49.94–84.50
Identical-image stage	Own-race	95.98	0.58	100	100	72.00–100
	Other-race	95.17	0.59	100	100	67.00–100
	Total	95.57	0.44	97.00	100	78.00–100
Novel-image stage	Own-race	54.93	1.19	57.00	57.00	23.00–94.00
	Other-race	44.73	1.16	45.00	43.00	17.00–83.00
	Total	49.83	0.98	50.00	45.00	25.00–78.50

Mean, standard error, median, mode and range of participants’ accuracy for stage 1 (Identical-Image) stage 2 (Novel-Image), and for two stages combined.

#### Overall accuracy across two stages

**Figure 4:**
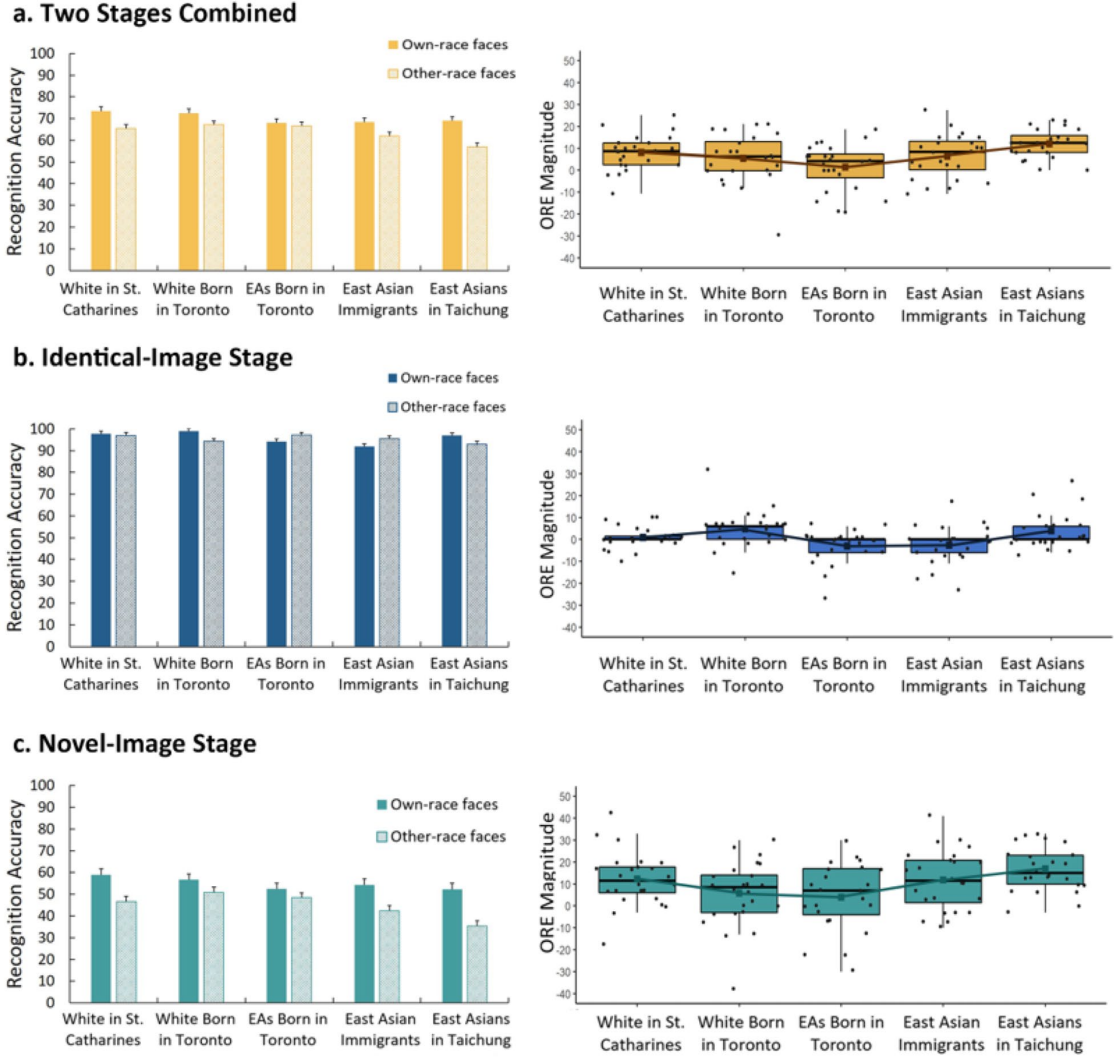
Percent correct for own- and other-race faces (left panels) and magnitude of the ORE (right panels) in 5 groups of participants for (**a**) two stages combined, (**b**) Identical-Image Stage, and (**c**) Novel-Image Stage. Midlines and cube marker inside the box represent medians and means. The bottom and top of boxes indicate the first and third quartiles.

Preliminary analyses revealed no significant effect of testing order (own- vs. other-race first), *p*s > .387. Therefore, data were collapsed across testing orders for further analyses. To examine whether overall recognition accuracy changed as a function of face race and participant group, we conducted a 2 × 5 mixed ANOVA, with race of faces (own-race vs. other-race) as a within-subject factor, and participant group (White in St. Catharines, White born in Toronto, East Asians born in Toronto, East Asian immigrants, East Asians in Taichung) as a between-subject factor. Significant effects were followed up with Bonferroni-corrected pairwise comparisons. The results revealed significant main effects of face race, *F*(1,115) = 59.70, *p* < .001, *η*_p_^2^ = 0.34, and participant group, *F*(1,115) = 3.86, *p* = .006, *η*_p_^2^ = 0.12. Accuracy was higher for own-race (*M* = 70.32, *SE* = 0.81) than other-race faces (*M* = 63.65, *SE* = 0.76). Pairwise comparisons revealed that the EA in Taichung group (*M* = 63.02, *SE* = 1.42) was significantly less accurate than the White in St. Catharines (*M* = 69.51, *SE* = 1.47) and White in Toronto group (*M* = 69.82, *SE* = 1.47; ps < .024), and no other groups were significantly different, *p*s > .289. Notably, we found a significant interaction between face race and participant group, *F*(4,115) = 4.05, *p* = .004, *η*_p_^2^ = 0.12. Follow-up tests suggest that whereas Toronto born Asians demonstrated no other-race effect, *p* = .523, the other four groups all showed less accurate recognition of other-race compared to own-race faces, *p*s < .029 (Fig. 4a, left panel). In addition to common frequentist statistics, we used Bayesian statistics^43^ to evaluate the strength of the evidence for the current pattern of results. Consistent with results from the classical analyses, a Bayesian 2 (face race) × 5 (participant group) mixed ANOVA found that the best performing model included both main effects and the interaction between the two factors. The data were 8.63 times more likely under this model than under the next-best performing model containing only the two main effects and no interaction. Follow-up Bayesian paired samples t-tests found overwhelming evidence for an other-race effect in the White in St. Catharines (BF_10_ = 486.47) and EA in Taichung groups (BF_10_ = 2.29e + 6), moderate evidence for an other-race effect in the EA immigrant group (BF_10_ = 9.47), anecdotal evidence for an other-race effect in the White in Toronto group (BF_10_ = 2.05), and moderate evidence of *no* other-race effect in the EA in Toronto group (BF_10_ = 0.26). Tables showing the full results of all Bayesian analyses reported in the paper can be found in the Supplementary Material.

Below we analyzed participants’ performance separately for the Identical-Image Stage (Stage 1) and Novel-Image Stage (Stage 2). In Stage 1, participants can rely on pictorial cues for recognition: They know which target will be presented and the learning and testing items are identical. In Stage 2, participants must recognize identity per se: They do not know which target will be presented and the testing items are new images of the learned identity. Recognition during Stage 2 requires participants to form generalizable representations of faces^44^, something that is impaired for other-race compared to own-race faces^23,24^.

#### Accuracy for identical-images stage

A 2 × 5 mixed ANOVA was conducted to examine how adults’ memory for own- and other-race faces is influenced by living environment. The main effect of face race was not significant, *F*(1,115) = 1.28, *p* = .261, *η*_p_^2^ = 0.01 (Own-race: *M* = 95.98, *SE* = 0.54; Other-race: *M* = 95.17, *SE* = 0.58)*.* The main effect of participant group was significant, *F*(4,115) = 2.75, *p* = .032, *η*_p_^2^ = 0.09. The EA immigrants (*M* = 93.25, *SE* = 0.97) had lower accuracy than White in St. Catharines (*M* = 97.40, *SE* = 0.97, *p*s < .030) and all other groups were not significantly different from each other, *p*s > ,143. The face race x participant group interaction was also significant, *F*(4,115) = 5.06, *p* = .001, *η*_p_^2^ = 0.15. Follow-up tests suggest that whereas White in Toronto and EAs in Taichung had an other-race effect, *p*s < .033, the other three groups did not have an other-race effect, *p*s > 0.064. Nonetheless, accuracy was high (> 91.96%) in each of the race x group cells (Fig. 4b, left panel). Somewhat consistent with results from the classical analyses, a Bayesian 2 (face race) × 5 (participant group) mixed ANOVA found that the best performing model included both main effects and the interaction between the two factors. The data were 13.54 times more likely under this model than under the next-best performing model. Follow-up Bayesian paired samples t-tests found anecdotal evidence for an other-race effect in the White in Toronto (BF_10_ = 3.66), EA in Taichung (BF_10_ = 1.87), and EA in Toronto groups (BF_10_ = 1.06), and anecdotal to moderate evidence of *no* other-race effect in the White in St. Catharines (BF_10_ = 0.285) and EA immigrant groups (BF_10_ = 0.58).

#### Accuracy for novel-images stage

We found significant main effects of face race, *F*(1,115) = 68.08, *p* < .001, *η*_p_^2^ = 0.37, and participant group, *F*(4,115) = 3.45, *p* = .011, *η*_p_^2^ = 0.11. Accuracy was higher for own- (*M* = 54.93, *SE* = 1.18) than other-race faces (*M* = 44.73, *SE* = 1.07). EAs in Taichung had lower recognition accuracy than White in St. Catharines, and White in Toronto group, *p*s < .037, and recognition accuracies in all other groups were not significantly different from each other, *p*s > .322. The predicted interaction between face race and participant group was significant, *F*(4,115) = 329.61, *p* = .008, *η*_p_^2^ = 0.11. Follow-up tests revealed that whereas White in Toronto and EAs born in Toronto demonstrated no differences in their recognition of own- and other-race faces, *p*s > 0.070, the other three groups all showed less accurate recognition of other- relative to own-race faces, *p*s < .001 (Fig. 4c, left panel). Consistent with results from the classical analyses, a Bayesian 2 (face race) × 5 (participant group) mixed ANOVA found that the best performing model included both main effects and the interaction between the two factors. The data were 4.71 times more likely under this model than under the next-best performing model containing only the two main effects and no interaction. Follow-up Bayesian paired samples t-tests found overwhelming evidence of an other-race effect in the White in St. Catharines (BF_10_ = 349.39) and EA in Taichung groups (BF_10_ = 5.04e + 5), very strong evidence for an other-race effect in the EA immigrant group (BF_10_ = 87.02), and anecdotal evidence for *no* other-race effect in the White in Toronto (BF_10_ = 0.99), and EA in Toronto groups (BF_10_ = 0.44).

#### Reaction time

To determine whether there was a speed/accuracy trade-off, we ran a 2 × 5 mixed analysis of variance (ANOVA) for mean response times for correct responses. This analysis was conducted separately for the identical-image, novel-image and the combination of the two stages. Response time was slower for other- than own-race faces for both overall task and the identical-image stage, *p*s < .017, but was comparable for the novel-image stage, *p* = .070. Therefore, lower accuracy for other-race faces cannot be attributed to other-race faces being reacted to faster than own-race faces. In addition, the Taichung EA and Toronto EA immigrant groups responded significantly slower than the other groups (St. Catharines White, Toronto White and Toronto born EA) across all stages, *p*s < .027. Therefore, lower accuracy in the two groups cannot be attributed to faster responding as compared to the other groups.

### Magnitude of the ORE, city of residence, and ethnicity

To directly examine how the magnitude of the ORE changes as a function of perceptual experience, we (1) calculated the size of the ORE by subtracting adults’ average recognition accuracy for other-race faces from their recognition accuracy for own-race faces and (2) ran three multiple linear regressions to predict adults’ magnitude of the ORE based on participant groups for Stage 1, Stage 2 and for two stages combined. The five groups of participants were dummy coded, with the Toronto born EA group, who has the smallest ORE, set as a reference. Preliminary analyses were performed to ensure there was no violation of the assumptions of normality and linearity. The analyses revealed that participant group significantly predicted the ORE magnitude for both stages combined, *F*(4, 119) = 4.05,* p* = .004, *R*^*2*^ = 0.12, *R*^*2*^_Adjusted_ = 0.09, for the identical-image stage, *F*(4, 119) = 5.02,* p* = .001, *R*^*2*^ = 0.15, *R*^*2*^_Adjusted_ = 0.12, and for the novel-image stage, *F*(4, 119) = 3.61,* p* = .004, *R*^*2*^ = 0.11, *R*^*2*^_Adjusted_ = 0.08.

For the two stages combined, the magnitude of the ORE was significantly larger in the St. Catharines White group (6.67 points, *t* = 2.44, *p* = .016) and the Taichung EA group (10.66 points, *t* = 3.90, *p* < .001) than the Toronto born EA group. The ORE magnitude in the Toronto White and Toronto EA immigrant group were not significantly different from Toronto-born EA group, *p*s > .067. For the identical-image stage, Toronto White (7.83 points, *t* = 3.44, *p* = .001) and Taichung EA group (7.00 points, *t* = 3.07, *p* = .003) had a greater ORE than the Toronto born EA group. The other two groups were not significantly different from the Toronto Born EA group in predicting their ORE magnitude, *p*s > .084. For the more difficult stage, where people needed to recognize novel images of learned faces, all groups’ ORE magnitudes were significantly greater than the Toronto born EA groups, *p*s < .046, except for the White in Toronto group, *t* = 0.49, *p* = .623. The results are summarized in Fig. 4 (right panel) and Table 3.

**Table 3:** Regression analysis summary for participant groups predicting adults’ magnitude of the other-race effect.

Predictor	Unstandardized Coefficients		Standardized Coefficients		*R*^2^	*F*	*p*
	B	SE	β	*p*			
**ORE magnitude for both stages as DV**					0.123	4.046	.004
EA in Toronto (reference)	1.40	2.73		.470			
White in St. Catharines	6.67	2.73	0.27*	.016			
White in Toronto	4.02	2.73	0.16	.144			
EA Immigrants	5.03	2.73	0.20	.068			
EA in Taichung	10.66	2.73	0.43***	.000			
**ORE magnitude for stage 1 as DV**					0.386	5.022	.001
EA in Toronto (reference)	− 3.04	2.28		.062			
White in St. Catharines	3.96	2.28	0.19	.085			
White in Toronto	7.83	2.28	0.37	.001			
EA Immigrants	0.46	2.28	0.02	.841			
EA in Taichung	7.00	2.28	0.33*	.003			
**ORE magnitude for stage 2 as DV**					0.334	3.611	.008
EA in Toronto (reference)	3.96	3.89		.152			
White in St. Catharines	8.29	2.75	0.24*	.035			
White in Toronto	1.92	3.89	0.06	.623			
EA Immigrants	7.88	3.89	0.23*	.045			
EA in Taichung	12.92	3.89	0.37***	.001			

*p < .05, ***p < .001.

### Magnitude of the ORE and reported interracial contact

**Figure 5:**
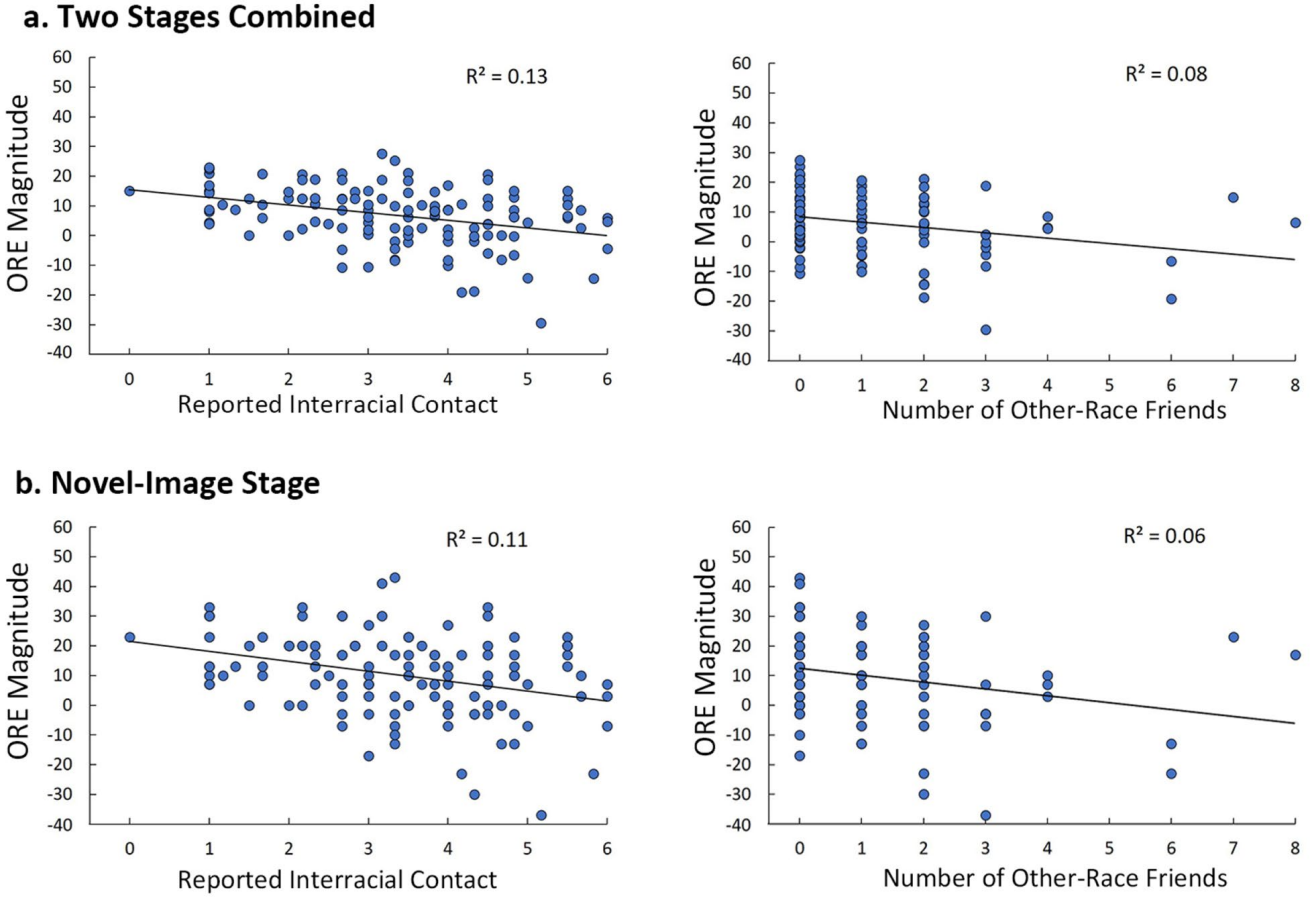
Reported interracial contact (left panel) and number of interracial friends (right panel) are negatively correlated with the magnitude of the ORE across both stages (**a**) and for the Novel-Image Stage (**b**). Dots indicate individual data of the magnitude of the other-race effect. Solid trendlines show lines of best fit.

The magnitude of the ORE in the novel-image stage and across both stages were negatively correlated with the other-race contact and number of other-race friends (two stages combined: contact: *r* = − 0.37, *p* < 0.001, friends: *r* = − 0.27, *p* = .003; Novel-Image Stage: contact: *r* = − 0.33, *p* < 0.001; friends: *r* = − 0.25, *p* = .006). The magnitude of the ORE for the Identical-Image Stage was only negatively correlated with other-race interactions (*r* = − 0.21, *p* = .022), but not correlated with the number of other-race friends (*r* = 0.12, *p* = .180). Scatterplots shows the significant results (Fig. 5).

## Discussion

Applying the novel Variability Face Memory Test (VFMT) that mimics the challenge of recognizing faces in daily life, we examined learning of own- versus other-race faces in White and East Asian adults who had different lifetime histories of interracial perceptual experience. Our cross-cultural study provides a systematic investigation of how perceptual experience shapes adults’ ability to learn own- and other-race faces across naturalistic facial variability in appearance and introduces a long overdue approach to the study of face learning. Taking advantage of the unique demographics in Toronto, one of the most multiracial areas in the world, this study further examined how multiracial immersion since birth sculpts the magnitude of the other-race effect in adulthood.

Our study provides direct support for the notion that the magnitude of the ORE in adulthood changes as a function of perceptual experience. The different ORE magnitude in the five groups of participants mirrors the different degree of perceptual experience that these adults have with other-race individuals, as reflected both in their group membership and personal interracial contact. The groups that showed the largest other-race effect were those that had the least interracial perceptual experience: Whites in St. Catharines and East Asians in Taichung. Across both stages of the memory tests, the magnitude of the other-race effect in these groups were no smaller than 6.66 points as compared to the Toronto-born East Asians, who consistently showed no other-race effect. The lack of the other-race effect is also evident in Toronto-born White participants when recognizing novel images of faces across variability in appearance. Collectively, these findings highlight the role of the racially homogeneous environment in enhancing the other-race effect, and the role of the multiracial environment in reducing or eliminating the other-race effect in adulthood.

The ORE has its origins in perceptual narrowing in infancy, a process whereby our broad perceptual system is tuned by experience to recognize faces from familiar categories at a cost of recognizing faces from less experienced face categories^45,46^. The emerging ORE might reflect a decline in the ability to discriminate among other-race faces^7^, a decline that can be postponed by experience^47–49^, or a failure to refine learning for other-race faces as learning of own-race faces improves^6^. Our finding that the other-race effect is diminished or absent in White and East Asian adults born in Toronto fits well with both accounts. Extensive early and continuous perceptual experience with own- and other-race faces, as a result of direct person-environment interaction since infancy, likely prevents the reduced sensitivity to other-race faces (i.e., diminishes perceptual narrowing) and facilitates the refinement of the other-race face representations (i.e., benefits perceptual learning of other-race faces), leading to the elimination of the other-race effect in the White and East Asian adults born in Toronto. Our finding is also consistent with the few studies that have examined other-race face processing in infants from multiracial environments^50,51^. Moreover, we found a robust ORE in the East Asian in Taichung group, and in the White in St. Catharines group (at least in the novel-image stage). This suggests that abundant own-race experiences and limited other-race experiences acquired during person-environment interaction likely set up the perceptual system in a way that is preferentially tuned for more frequently encountered faces, namely own-race faces, resulting in enhanced recognition of own-race faces compared to other-race faces.

Convergent evidence suggests that the timing of other-race exposure influences the magnitude of the other-race effect in adulthood, as the perceptual system seems to be more flexible in infancy and childhood than in adulthood^37,39,40^. Other-race face recognition is improved by childhood but not adult contact^37^. Whereas East Asians who immigrated to Canada during infancy and childhood showed no other-race effect in adulthood, those who immigrated during adolescence and adulthood continued to show long-lasting disadvantage in recognizing other-race faces in adulthood. Here, we found that East Asian immigrants consistently demonstrated an ORE in adulthood (in the novel-image stage and two stages combined). Considering that nearly 90% of our East Asian immigrant sample moved to Toronto when they were adults, it is reasonable to argue that the ORE might be more difficult to change in adulthood than in childhood. However, the exact developmental window during which exposure to new categories of faces can shape the system, and how the quality and quantity of perceptual experience shapes the trajectory of other-race face processing, deserves further investigation. Bronfenbrenner’s influential ecological systems theory^52^ suggests that individuals continuously interact with both micro- and macro-levels of direct and indirect environment during development. It is therefore important to examine in future studies how different interactions with each layer of the living environment (e.g., home, school, neighborhood, community, and society) give rise to different processing of own- and other-race faces during development.

The Bayesian analyses revealed that there is strong evidence for an other-race effect when recognition is assessed across both stages or novel-image stage (for people living in racially homogeneous environment, and EA immigrants), whereas the evidence for an other-race effect is weak or non-existent for the identical images stage. This is an important finding as it suggests that the other-race effect in the real world can be greatly underestimated when each identity is represented by a single image or two similar images. Both own-and other-race faces in our daily life vary naturally in appearance due to lighting, expression, viewing angles, hairstyle and make-up. Social interactions often require one to form robust facial representations that can be generalized to recognize novel instances of faces despite a wide range of variability in facial appearance. Our study therefore highlights the necessity of incorporating naturalistic within-person variability and investigating how perceptual experience shapes the utilization of pictorial versus structural cues, in own- versus other-race recognition in future research. We also note that recognition accuracies for both own-race (70.3%) and other-race faces (63.7%) in our study are slightly lower than recognition accuracy for the Cambridge Face Memory Test (CFMT) in McKone and colleagues’ study^20^ (76% and 66% for own- and other-race faces), where a noise stage was included. In future studies, it would be interesting to investigate the relationship between memory performance in the Variability Face Memory Test (VFMT) and performance in the Cambridge Face Memory Test, as well as in other face memory tests (e.g., old/new face recognition tests). It is also interesting to examine in future studies how face memory decays with the increase of the number of faces learned and tested in the Variability Face Memory Test.

Our research has several limitations. First, only female celebrities’ faces were used. It remains unclear whether experience shapes the other-race effect differently for male and female faces. Additionally, celebrities might vary their appearance more than the typical population and so it would be important to determine whether our findings generalize to the learning of non-celebrities’ faces. Next, social cognitive factors such as social categorization and motivated individuation contribute to the other-race effect^9^.The current study did not examine how these factors shape the magnitude of the other-race effect, yet it is a question worthy of further examination.

Nevertheless, our study provides novel insights about the magnitude of the ORE when in the context of within-person variability in appearance, a condition that more closely mimics the challenge of recognizing faces in daily life. It also provides new evidence about how the ORE is shaped by experience, as a result of direct person-environment interaction. Our study suggests that different ORE magnitudes in individuals who live in different cities with varying ethnic compositions might reflect an adaptive function of our perceptual system in response to the living environment. In addition, our novel VFMT builds on the widely cited CFMT (810 citations in the past 15 years) and provides a robust tool to examine face learning and recognition of images that incorporate naturalistic within-person variability in appearance—a task that is available for testing various populations (e.g., children, older adults, professionals tasked with judging photo ID) and is essential for moving both theory^21^ and applied face perception research^53^ forward.

## Supplementary Information

Supplementary Information.

## Data Availability

All data have been made publicly available via the Open Science Framework (OSF) and can be accessed at: anonymized link.
