# Supplementary material for: Multi-cultural cities reduce disadvantages in recognizing naturalistic images of other-race faces: evidence from a novel face learning task
Source: Sci Rep. 2022 May 27;12:8950. doi: 10.1038/s41598-022-11550-9 (PMC9142532; doi:10.1038/s41598-022-11550-9)
Supplement: Supplementary file 1 — Supplementary Information. [file 41598_2022_11550_MOESM1_ESM.docx]

**SUPPLEMENTARY MATERIAL**

**Multi-cultural cities reduce disadvantages in recognizing naturalistic images of other-race faces: Evidence from a novel face learning task**

*Scientific Reports*

Date of this version: January 2022

List of Supplementary Material

SM1: White and East Asian Celebrities’ Names

SM2: Familiarity and Racial Contact Questionnaire

SM3: Results of Bayesian Analyses (Table 1a-3c)

***SM1. White and East Asian Celebrities’ Names***

White Celebrities

| Holly Willoughby | Elinor Crawley | Frida Gustavsson | Snejana Onopka |
| --- | --- | --- | --- |
| Fearne Cotton | Elsa Hosk | Emily Beecham | Coco Rocha |
| Alex Jones | Gabriella Wilde | Toni Garrn | Kate Grigorieva |
| Stacey Dooley | Kimberley Nixon | Laura Whitmore | Elen Rhys |
| Angela Scanlon | Isabelle Ithurburu | Roisin O | Seana kerslake |
| Vogue Williams | Andreja Pejic | Kasia Struss | Sallie Harmsen |
| Anais Gallagher | Sanne Langelaar | Bracha van Doesburgh | Geraldine Kemper |
| Edie Campbell | Constance Jablonski | Charlie Murphy | Lauren Verster |
| Tess Milne | Lily Donaldson | Mischa Barton | Katja Herbers |
| karen Hassan | Enikő Mihalik | Helen Skelton | Abbey Hoes |
| Valery Kaufman | Pip Pellens | Nina Agdal | loes haverkort |
| Sara Forestire | Sigrid Agren | Freja Beha Erichsen | Jelka van Houten |
| Sarah Greene | Hanna Verboom | Perdita Weeks | Amy Shiels |

East Asian Celebrities

| Yuriko Yoshitaka | Koyuki | Mirai Shida | Kanako Enomoto |
| --- | --- | --- | --- |
| Erika Toda | Mao Inoue | Mirei Kiritani | Mizuki Fukumura |
| Keiko Kitagawa | Mariya Nishiuchi | Yoko Maki | Kazue Fukiishi |
| Satomi Ishihara | Aoi Miyazaki | Kasumi Arimura | Seika Furuhata |
| Nagasawa Masami | Ryōko Hirosue | Tao Okamoto | Maki Goto |
| Kou Shibasaki | Emi Takei | Chizuru Ikewaki | Rika Izumi |
| Maki Horikita | Mikako Tabe | Anna Nagata | Mari Hoshino |
| Haruka Ayase | Haruna Kawaguchi | Yūko Takeuchi | Yui Ichikawa |
| Rinko Kikuchi | Honoka Yahagi | Natsumi Abe | Riho Iida |
| Yui Aragaki | Saki Aibu | Rika Adachi | Mayuko Fukuda |
| Yukie Nakama | Aya Omasa | Rina Aizawa | Ayumi Ito |
| Kyoko Fukada | Sasaki Nozomi | Nao Asahi | Chao Chen |
| Aya Ueto | Juri Ueno | Mew Azama | Zi Cai |

note. Blue boxes indicate celebrities whose faces were used as target faces. The rest were used as distractor faces.

***SM2. Racial Contact & Familiarity Questionnaire***

**Racial Interaction Questionnaire**

**1. Your birthdate: ______________**

**2. Would you generally self-identify as:**

Male___

Female___

Other___

Prefer not to answer ___

**3. What is your ethnicity?**

☐Aboriginal

☐Arab

☐Black

☐Filipino

☐Chinese

☐Japanese

☐Korean

☐Latin American

☐South Asian (e.g., East Indian, Pakistani, Sri Lankan, etc.)

☐Southeast Asian (e.g., Vietnamese, Cambodian, Malaysian, Laotian, etc.)

☐West Asian (e.g., Iranian, Afghan, etc.)

☐White

☐Other: ______

**4. In which country, and city were you born? _________________________**

**5. How long have you been in the country you are living in now? ______________**

**6. Please list all the countries you have lived in, the length of time in each and your approximate age while you were living there.**

Location Duration (approx.) Your Age when there (approx.)

**______________ ________________ ____________________**

**______________ ________________ ____________________**

**______________ ________________ ____________________**

**7. In which country was your biological mother born?** ____________________

**What is her ethnicity?**  ____________________

**In which country was your father born?** ____________________

**What is his ethnicity?**  ____________________

**8. Do you have any other relatives who are members of other ethnic or racial groups?**

**(by birth or by marriage?)**

**YES** ________ **NO** ________

If so, please list:

Their Relationship to you How often do you see them (approx.)?

Ethnicity *(aunt, cousin etc)*

|  |  | *Weekly* | *Monthly* | *Yearly* | *Less than yearly* |
| --- | --- | --- | --- | --- | --- |
|  |  |  |  |  |  |
|  |  |  |  |  |  |
|  |  |  |  |  |  |
|  |  |  |  |  |  |

9. **Have you ever lived with people from other ethnic groups?**

**YES** ________ **NO** ________

If so, please list:

Their Length Your age when you moved in with

Ethnicity of cohabitation them (approximately)

**______________ ________________ ____________________**

**______________ ________________ ____________________**

**______________ ________________ ____________________**

**______________ ________________ ____________________**

**10.** **In the following section, we would like you to indicate how well the following statements represent the type of interactions you have with Asian and White/Caucasian people.**

Please indicate the extent to which each statement represents your interactions by circling the number which best represents your opinion.

**Very strongly Strongly Disagree Agree Strongly Very strongly**

**Disagree Disagree Agree Agree**

**1 2 3 4 5 6**

| 1 | I know lots of Asian people | 1 | 2 | 3 | 4 | 5 | 6 |
| --- | --- | --- | --- | --- | --- | --- | --- |
| 2 | I interact with White/Caucasian people during recreational periods | 1 | 2 | 3 | 4 | 5 | 6 |
| 3 | I live, or have lived in an area where I interact with White/Caucasian people | 1 | 2 | 3 | 4 | 5 | 6 |
| 4 | I live, or have lived in an area where I interact with Asian people | 1 | 2 | 3 | 4 | 5 | 6 |
| 5 | I interact with Asian people during recreational periods | 1 | 2 | 3 | 4 | 5 | 6 |
| 6 | I interact with White/Caucasian people on a daily basis | 1 | 2 | 3 | 4 | 5 | 6 |
| 7 | I socialize a lot with White/Caucasian people | 1 | 2 | 3 | 4 | 5 | 6 |
| 8 | I went to a high school where I interacted with Asian students | 1 | 2 | 3 | 4 | 5 | 6 |
| 9 | I socialize a lot with Asian people | 1 | 2 | 3 | 4 | 5 | 6 |
| 10 | I know lots of White/Caucasian people | 1 | 2 | 3 | 4 | 5 | 6 |
| 11 | I interact with Asian people on a daily basis | 1 | 2 | 3 | 4 | 5 | 6 |
| 12 | I went to a high school where I interacted with White/Caucasian students | 1 | 2 | 3 | 4 | 5 | 6 |

11. **Think of up to 10 friends with whom you spend the most time. Of these 10 friends:**

How many are Caucasian? _______

How many are East Asians? _______

How many are any other race outside of Caucasian and East Asians? _______

**12. Indicate your response by marking the point on the scale.**

Please rate your amount of interaction with **White/Caucasian** individuals in this country in general.

**1 2 3 4 5 6 7**

***Little A lot***

***or none***

Please rate your amount of interaction with **East Asian** individuals in this country in general.

**1 2 3 4 5 6 7**

***Little A lot***

***or none***

**Familiarity Information**

Were any of the White and East Asian faces in the experiment familiar to you? Please circle

Yes / No

If yes, please indicate the name/s* of the individual/s that you recognised:

______________________________________________________________________________

______________________________________________________________________________

*If you can't recall a name then please write down other information related to that person (e.g. actor from a Harry Potter film)

______________________________________________________________________________

______________________________________________________________________________

______________________________________________________________________________

______________________________________________________________________________

***SM3. Results of Bayesian Analyses***

***Overall accuracy across two stages***

Supplementary Table 1a. Model Comparison

| **Models** | **P(M)** | **P(M\|data)** | **BF_M_** | **BF_01_** | **error %** |
| --- | --- | --- | --- | --- | --- |
| Face Race + Participant Group + Face Race ✻ Participant Group | 0.2 | 0.882 | 29.964 | 1.000 |  |
| Face Race + Participant Group | 0.2 | 0.102 | 0.456 | 8.630 | 2.338 |
| Face Race | 0.2 | 0.016 | 0.063 | 56.782 | 2.373 |
| Participant Group | 0.2 | 1.836e-10 | 7.343e-10 | 4.806e+9 | 1.987 |
| Null model (incl. subject) | 0.2 | 3.967e-11 | 1.587e-10 | 2.224e+10 | 1.946 |
|  | | | | | |
| *Note.*  All models include subject. | | | | | |

Supplementary Table 1b. Analysis of Effects

| **Effects** | **P(incl)** | **P(excl)** | **P(incl\|data)** | **P(excl\|data)** | **BF_incl_** |
| --- | --- | --- | --- | --- | --- |
| Face Race | 0.6 | 0.4 | 1.000 | 2.178e-10 | 3.061e+9 |
| Participant Group | 0.6 | 0.4 | 0.985 | 0.015 | 43.341 |
| Face Race ✻ Participant Group | 0.2 | 0.8 | 0.885 | 0.115 | 30.657 |

Supplementary Table 1c. Paired-samples t-tests

| **Participant Group** | **BF_10_** | **error %** |
| --- | --- | --- |
| White in St. Catharines | 486.471 | 1.205e-5 |
| White in Toronto | 2.051 | 0.003 |
| EA in Toronto | 0.260 | 0.036 |
| EA Immigrants | 9.469 | 3.458e-4 |
| EA in Taichung | 2.295e+6 | 1.289e-10 |

***Accuracy for Identical-Images Stage (Stage 1)***

Supplementary Table 2a. Model Comparisons

| **Models** | **P(M)** | **P(M\|data)** | **BF_M_** | **BF_01_** | **error %** |
| --- | --- | --- | --- | --- | --- |
| Face Race + Participant Group + Face Race ✻ Participant Group | 0.2 | 0.855 | 23.575 | 1.000 |  |
| Null model (incl. subject) | 0.2 | 0.063 | 0.270 | 13.538 | 1.972 |
| Participant Group | 0.2 | 0.053 | 0.225 | 16.083 | 2.024 |
| Face Race | 0.2 | 0.016 | 0.064 | 54.495 | 2.654 |
| Face Race + Participant Group | 0.2 | 0.013 | 0.053 | 65.460 | 2.224 |
|  | | | | | |
| *Note.*  All models include subject. | | | | | |

Supplementary Table 2b. Analysis of Effects

| **Effects** | **P(incl)** | **P(excl)** | **P(incl\|data)** | **P(excl\|data)** | **BF_incl_** |
| --- | --- | --- | --- | --- | --- |
| Face Race | 0.6 | 0.4 | 0.881 | 0.119 | 4.940 |
| Participant Group | 0.6 | 0.4 | 0.920 | 0.080 | 7.669 |
| Face Race ✻ Participant Group | 0.2 | 0.8 | 0.852 | 0.148 | 23.056 |

Supplementary Table 2c. Paired-samples t-tests

| **Participant Group** | **BF_10_** | **error %** |
| --- | --- | --- |
| White in St. Catharines | 0.285 | 0.036 |
| White in Toronto | 3.664 | 4.072e-4 |
| EA in Toronto | 1.065 | 1.014e-4 |
| EA Immigrants | 0.578 | 1.128e-4 |
| EA in Taichung | 1.871 | 0.004 |

***Accuracy for Novel-Images Stage (Stage 2)***

Supplementary Table 3a. Model Comparisons

| **Models** | **P(M)** | **P(M\|data)** | **BF_M_** | **BF_01_** | **error %** |
| --- | --- | --- | --- | --- | --- |
| Face Race + Participant Group + Face Race ✻ Participant Group | 0.2 | 0.787 | 14.818 | 1.000 |  |
| Face Race + Participant Group | 0.2 | 0.167 | 0.803 | 4.709 | 1.903 |
| Face Race | 0.2 | 0.045 | 0.190 | 17.362 | 1.812 |
| Participant Group | 0.2 | 1.747e-11 | 6.989e-11 | 4.507e+10 | 1.451 |
| Null model (incl. subject) | 0.2 | 6.825e-12 | 2.730e-11 | 1.154e+11 | 1.348 |
|  | | | | | |
| *Note.*  All models include subject. | | | | | |

Supplementary Table 3b. Analysis of Effects

| **Effects** | **P(incl)** | **P(excl)** | **P(incl\|data)** | **P(excl\|data)** | **BF_incl_** |
| --- | --- | --- | --- | --- | --- |
| Face Race | 0.6 | 0.4 | 1.000 | 2.461e-11 | 2.709e+10 |
| Participant Group | 0.6 | 0.4 | 0.953 | 0.047 | 13.591 |
| Face Race ✻ Participant Group | 0.2 | 0.8 | 0.781 | 0.219 | 14.236 |

Supplementary Table 3c. Paired-samples t-tests

| **Participant Group** | **BF_10_** | **error %** |
| --- | --- | --- |
| White in St. Catharines | 349.387 | 1.226e-5 |
| White in Toronto | 0.999 | 1.026e-4 |
| EA in Toronto | 0.443 | 1.180e-4 |
| EA Immigrants | 87.015 | 6.588e-5 |
| EA in Taichung | 5.042e+5 | 8.004e-10 |
